# Are Self-Efficacy Gains of University Students in Adapted Physical Activity Influenced by Online Teaching Derived From the COVID-19 Pandemic?

**DOI:** 10.3389/fpsyg.2021.654157

**Published:** 2021-04-09

**Authors:** Alba Roldan, Raul Reina

**Affiliations:** Department of Sport Sciences, Sports Research Centre, Miguel Hernández University, Elche, Spain

**Keywords:** inclusion, special education needs, para-sport, disability, higher education, distance learning

## Abstract

Due to the lockdown caused by the COVID-19 pandemic, e-learning suddenly spread to different levels of education, including university. In Spain, students of sports sciences are prepared during a 4-year study program to work in different areas (including physical education) and with different populations (including people with disabilities). The aims of this study were (1) to assess the effect of pandemic-driven online teaching on self-efficacy (SE) for the inclusion of people with disabilities in a group of university students enrolled in a compulsory course on adapted physical activity (APA); (2) compare the gains from SE before and after the APA course with a sample of students who followed the same course before the pandemic; and (3) explore the influence on SE scores according to three demographic variables: gender, previous SE training, and previous experience with people with disabilities. The study involved a sample of 124 university students (22.1 ± 2.6 years), distributed into two groups: prepandemic (*n* = 86) and pandemic (*n* = 38). They voluntarily completed the Spanish version of the Scale of Self-Efficacy of Physical Education Teachers of Physical Education toward Children with Disabilities, obtaining pre- and postcourse measurements. Due to the sudden lockdown, two-thirds of the pandemic course was taught online, and changes in the teaching strategies and tasks had to be made. Similar gains were obtained in both groups for the three subscales of the SE scale (*p* < 0.001, large effect sizes): intellectual, physical, and visual disability. No significant differences were found for comparisons between groups and the interaction effect of the course taught, nor for the three demographic co-variables. This study shows that teaching strategies that encourage student participation and reflections on learning increase student SE, regardless of the teaching format (i.e., face-to-face vs. online teaching). Moreover, the gains in SE are invariable to demographic co-variables.

## Introduction

The World Health Organization (WHO) has declared a world pandemic due to severe acute respiratory syndrome coronavirus 2 (SARS-CoV-2), popularly known as coronavirus disease 2019 (COVID-19). In response, most affected countries have enacted measures involving compulsory confinement and restrictions on free movement, which likely influence citizens’ lifestyles (López-Bueno et al., 2020). Governments have implemented combinations of “lockdown” measures with varying degrees of stringency, so public health recommendations and governmental measures have enforced numerous restrictions on daily living, including social distancing, isolation, and home confinement (Ammar et al., 2020). In Spain, the national government declared the state of alert (BOE, 2020), where many of these measures were mandatory for all the population. In consequence, Spanish universities not only had to adapt to the changes derived from the causes of the pandemic but also had to face a new methodological model—e-learning teaching—which not all teachers and students were trained for (Alemany-Arrebola et al., 2020).

Before this pandemic scenario, evidence corroborated the differences between face-to-face and virtual classroom contexts because of the “qualities and characteristics of the teaching/learning experience” (Rice, 2006, p. 432–433). In the regular prepandemic face-to-face classrooms, learning and growth in students have been found to closely correlate with teachers’ self-efficacy (SE; Goddard et al., 2000). However, throughout the coronavirus pandemic, teachers have had to adjust their teaching techniques for classes that take place online. During this adjustment process, teachers have faced tremendous challenges, especially in the first weeks of the confinement, when they had to manage a great deal of work and the accelerated learning of new technological skills to accomplish their teaching (Allen et al., 2020). In short, teachers may have been confident in how to teach their content in the classroom; however, this confidence may differ when teaching virtually, with little to no preparation.

**Figure 1:**
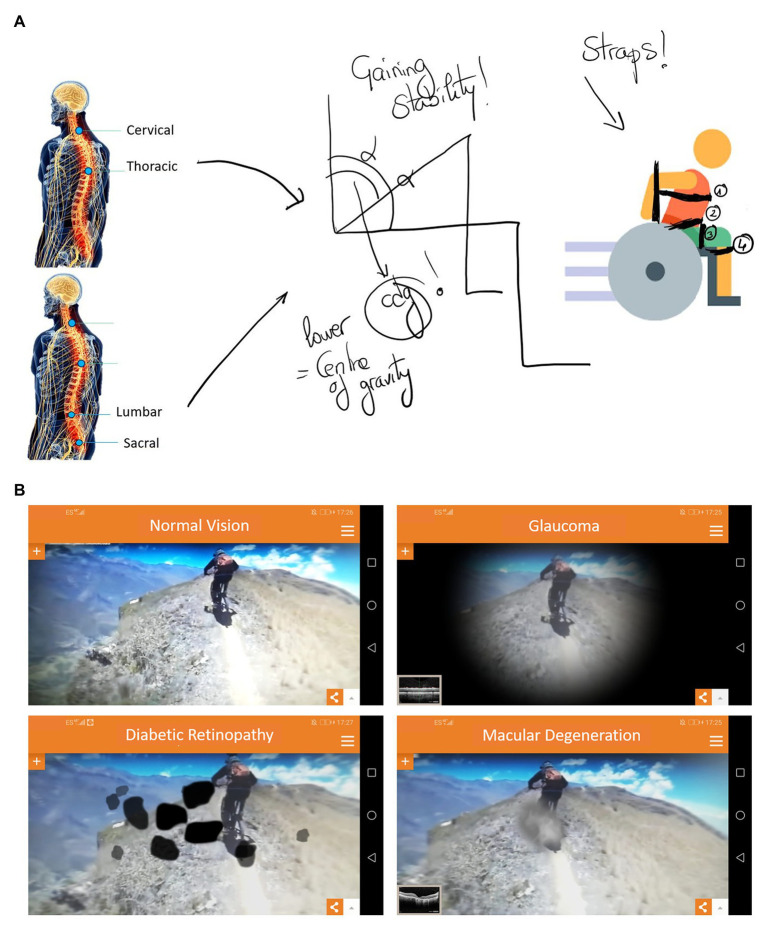
**(A)** Wheelchair straps for improving sitting stability explained using a graphic tablet. **(B)** ViaOpta Simulator (Novartis™) app for simulating visual impairments in an open context.

SE is an effective predictor of performance (Bandura, 1991) and has either a direct or indirect influence on teachers’ behavior (Woods and Rhoades, 2013). Bandura (1991, p. 257) defined SE as “people’s beliefs about their capability to exercise control over their level of functioning and over events that affect their lives.” The SE theory postulates that individuals’ beliefs about their capacity to perform a task successfully influence their behavior, thoughts, and actions, providing an adequate theoretical framework for assessing self-confidence (Bandura, 1994). In pedagogical terms, teachers’ SE has been defined as the educator’s perception of their capability to facilitate students’ learning of knowledge, values, and behaviors successfully (Zach et al., 2012; Block et al., 2013).

In 2015, the United Nations established Sustainable Development Goal Four (SDG-4), which focuses on quality education, aiming to ensure that qualified, professionally trained, motivated, and well-supported teachers are available to all students (UNESCO, 2015), highlighting the role that teachers play in the complex education process. However, when talking about students with disabilities, studies in inclusive education have demonstrated that physical education (PE) teachers present negative attitudes toward their inclusion, driven by a poor SE in meeting students’ needs (Hodge and Elliott, 2013; Beamer and Yun, 2014; Reina et al., 2019a). This lack or poor professional preparation for facing the specific demands of people with disabilities has been identified as a barrier for promoting participation in physical activity and sports activities, including those with intellectual (Bodde and Seo, 2009; van Schijndel-Speet et al., 2014; Taliaferro and Hammond, 2016), physical (Buffart et al., 2009; Williams et al., 2014), visual (Jaarsma et al., 2014; Griffin et al., 2016), and hearing (Kurková, 2016) disabilities.

Three factors have been identified as contributing to the SE of a general teacher, namely efficacy in the use of inclusive instruction, efficacy in designing cooperation activities, and efficacy in the management of behavior (Sharma et al., 2011; Savolainen et al., 2012). However, while PE teachers share these SE attributes (Hutzler et al., 2019), sports and many physical activities must be dealt with in open-spaced environments. In consequence, excessive movement of the people involved, the special and variety of equipment used, challenging and interactive tasks, and exhaustive or health-related exercises add challenges to those encountered by the general teacher. These challenges have an impact not only on behavioral issues but also on safety and control issues when teaching people with disabilities (Morley et al., 2005). Besides, the pandemic led to educational and professional situations of “absent presence” and “touchless” (Varea and González-Calvo, 2020), and some preliminary results with preservice PE teachers report that missing the physical contact with students provokes a feeling that the subject of PE is losing its identity.

In Spain, the university studies in Physical Activity and Sports Sciences are a 4-year program, comprising a total of 240 ECTS (European Credit Transfer and Accumulation System) credits in the curriculum. These university studies allow alumni access to the labor market as PE teachers, among other options (Del Villar, 2006). Although there is a European standard for delivering adapted physical activities (APAs; Kudláček et al., 2010), the topic has a noticeable heterogeneity across sports sciences and physical activity curriculums in higher education in Spain. Roldan and Reina (2018) described a set of 11 bullet points that APA professionals should acquire for delivering physical activity and sports for people with disabilities (PwD), including basic features of the population and the implications on their daily life activities, specific communication skills, adapted equipment, task design for ensuring active and inclusive participation, sharing examples of good practice, awareness activities, specific regulations, and the basis of Paralympic and parasports, among others. In this context, Jimenez-Monteagudo (2016) studied the competencies that physical activity and sports science university-level students should acquire in APA, highlighting the relevance of contact with people with disabilities together with a critical reflection about their learning process for adapting their acquired professional skills to a diverse population.

The SE theory has proved successful in measuring both preservice (e.g., Alhumaid et al., 2020) and in-service (e.g., Reina et al., 2019a,b) teachers’ self-efficacy toward inclusive education. Considering that one of the main professional paths in university-level studies in sports sciences and physical education is teaching in PE (Del Villar, 2006; Gambau-Pinasa, 2014), this study aimed (1) to assess the effect of the pandemic-driven online teaching on SE toward the inclusion of PwD in a group of university students enrolled in a compulsory APA course; (2) to compare the pre- vs. postcourse SE scores with a sample of students that followed the same subject, without pandemic, at the same university, and with the same professors; and (3) to explore the influence on SE scores by three demographic variables, i.e., sex, previous training in APA, and previous experiences with PwD.

## Materials and Methods

### Participants

A sample of 124 (69 men and 55 women) physical activity and sports sciences university students from a south-east Spanish university took part in this study. When data collections took place, all the students were enrolled in the course on APA. The sample was divided into two subgroups according to whether it was a pandemic year or not: (1) prepandemic in the academic year 2017–2018 and (2) pandemic group in the academic year 2019–2020. The APA course always covers 4 months, from February to May. The pandemic group received 1-month face-to-face teaching moving quickly to online teaching at the beginning of March 2020. Table 1 shows the demographics reported for the entire sample and both subgroups, considering their age, gender, previous training in APA, and previous experiences and/or contact with PwD.

**Table 1:** Sample demographics.

	Previous pandemic	COVID-19 pandemic	Overall sample
Number	86	38	124
Age	22.1 ± 2.6	22.1 ± 2.2	22.1 ± 2.5
**Gender**			
Male	55.8%	55.3%	55.6%
Female	44.2%	44.7%	44.4%
**Previous training in APA**			
Yes	28.8%	34.3%	30.6%
No	71.2%	65.7%	69.4%
**Experience with PwD**			
Yes	43.1%	44.1%	43.4%
No	56.9%	55.9%	56.6%

APA, adapted physical activity; PwD, people with disabilities.

All the students participated voluntarily since the fulfillment of the pre- and postcourse measurement was not mandatory, and informed consent was provided before the administration of questionnaires. Taking into account the total number of students enrolled in the courses of the two academic years (*n* = 254), the response ratio was 63.8 and 32.8% for the prepandemic and during pandemic groups, respectively, also considering their attendance to over 70% of the lessons described in the procedure’s subsection.

### Measures

To evaluate the effect of the university course on student’s SE, the Self-Efficacy Scale for Physical Education Teacher Education Majors toward Children with Disabilities (SE-PETE-D) was used. This questionnaire was created and validated by Block et al. (2013) in English and it was adapted (Reina et al., 2016) and validated (Reina et al., 2019c) to the Spanish context [Escala de Autoeficacia en Profesores de Educación Física hacia Alumnos con Discapacidad (EA-PEF-AD)]. The questionnaire begins with a general introduction to Bandura’s SE theory and general guidance for using the rating scale to answer the questions. A section for self-reported demographics is also included (i.e., gender, age, previous training on APA, and previous experiences and/or contact with PwD, both in general or sports-specific settings). The questionnaire includes three vignettes describing a child with an intellectual disability, a physical disability, and a visual impairment, following a set of questions relating to fitness testing, teaching sport skills, and organizing the actual playing of a sport (i.e., intellectual = 11, physical = 12, and visual = 10 questions). These questions measure how competent the respondent feels in each category, measured by a Likert scale from 1 (no confidence) to 5 (complete confidence). In consequence, higher scores on these three subscales mean higher perceived competence to accommodate or include a PwD in PE. In the first sessions of both courses, just before any content explanation by the professors, students were invited to fulfill the SE-PETE-D survey, which they did and returned anonymously. However, for the postcourse measurements, the prepandemic group followed the same process, but the group affected by the pandemic completed the survey online using Google Forms dataset (Alphabet Inc., Mountain View, CA, United States). For matching the pre- and postcourse measures, every student introduced a four-digit code obtained from the last four numbers of their smartphone, a datum that is not accessible to the professors/researchers. To use this tool with in-service and preservice physical educators, approval by the ethics committee of the researchers’ university was obtained previously to data collections (DPS.RRV01.15).

### Intervention — APA Course Description

The course on APA of the Miguel Hernández University (Spain) is a mandatory course of 7.5 ECTS credits in the second-last year of the academic curriculum in Physical Activity and Sports Sciences. This number of credits represents 25% of a semester, 12.5% of an academic year, and 3.13% of the 4-year curriculum. Since 2017, the course has been delivered consistently by two professors, i.e., a male full professor (43 years old, teaching APA at university level since 2003, professional experience in APA since 1996) and a female assistant professor (37 years old, teaching APA at university level since 2016, professional experience in APA since 2010).

The syllabus of this course includes 4.5 ECTS of theoretical and 3 ECTS of practical lessons, representing the larger mandatory course on this topic among all Spanish universities (regular range: 4.5 ECTS). The students’ schedule is distributed as follows: 75 h of face-to-face training with the professors, 23.5 h of team-working, and 89 h of self-paced workload. The course contributes to the achievement of 6/12 and 5/19 of the general and specific professional skills of the university curriculum degree. For this purpose, the course syllabus contains 10 course-specific skills and has the following aims: (1) to identify barriers to and facilitators for accessing physical activity and parasports by PwD; (2) to correctly use the terminology about different groups and type of disabilities; (3) to know the benefits that physical activity and sports practice bring to PwD; (4) to analyze the contextual factors for delivering adapted programs according to specific needs; (5) to know the main modifications made in parasports and Paralympic sports; (6) to have indirect and direct contact with PwD; (7) to understand the basis of the Paralympic classification, including observation skills for linking eligible impairments and activity limitation; (8) to design inclusive strategies in physical activity and sports; (9) to develop home-made adapted materials and resources; (10) to accept the differences and diversity of PwD; and (11) to handle different scales for assessing mobility, function, and health in PwD.

For 15 weeks (i.e., from February to May), the course is delivered in four sessions per week, with a total of 3.5 h with the whole group and 1.5 h for practical content in small groups at sports facilities. The content of the course is organized into five educational units and 12 themes; the content and timing are described in Table 2. Attendance to the lessons is not mandatory, except one single day (3.5 h) where students deliver a teamwork-assigned task (i.e., inclusive games) with real para-athletes with intellectual or physical disabilities.

**Table 2:** Structure of the course syllabus.

Educational unit		Theme		Classroom sessions	Sports hall sessions
1	Introduction to APA and para-sports	1.1.	Conceptualization of disability, areas for intervention in APA, and parasports organization	3 (4.5 h)	1 (1.5 h)
		1.2.	Programs design in APA and parasport	2 (3 h)	–
2	Neurodevelopmental disorders	2.1.	Intellectual disability, ASD^*^, and ADHD^*^	4 (4 h)	1 (1.5 h)
		2.2.	Sports for people with intellectual disabilities. VIRTUS and Special Olympics	2 (2 h)	2 (3 h)
3	Physical disability	3.1.	Spinal cord injury, spina bifida, cerebral palsy, stroke, TBI, amputation, short stature, muscle dystrophy, and multiple sclerosis^*^	6 (6.5 h)	2 (3 h)
		3.2.	Classification in Paralympic sports	3 (3 h)	1 (1.5 h)
		3.3.	Sports for people with physical impairments	3 (3.5 h)	2 (3 h)
4	Sensory impairments	4.1.	Visual impairment^*^	3 (3.5 h)	1 (1.5 h)
		4.2.	Hearing impairment^*^	2 (2.5 h)	1 (1.5 h)
		4.3.	Sports for people with visual and hearing impairments	3 (3.5 h)	2 (3 h)
5	Accessibility in education and community settings	5.1.	Inclusion in physical education and educational settings	3 (3.5 h)	1 (1.5 h)
		5.2.	Accessibility in sports facilities	3 (3.5 h)	1 (1.5 h)

ASD, autism spectrum disorder; ADHD, attention-deficit hyperactivity disorder; TBI, traumatic brain injury.

* Includes a general conceptualization of the disability/impairment, the main activity limitations and/or restrictions to participating in physical activity and parasports, and guidelines for delivering program/inclusion.

With the declaration of the national state of alert by the SARS-CoV-2 pandemic (BOE, 2020), all universities in Spain moved to an online teaching model. In a few days, all the programmed activities were reconsidered and adapted to the new situation that continued until the end of the semester (i.e., last week of May 2020). All the online sessions were delivered using Google Meet (Alphabet Inc., Mountain View, CA, United States), a meeting app that was immediately implemented in the virtual teaching platform with open access for professors and students. Table 3 shows the planned teaching activities included in the course and the differences because of the pandemic.

**Table 3:** Differences for teaching delivery provoked by the SARS-CoV-2 pandemic.

Teaching strategies	Nonpandemic year	Pandemic year	Outcomes from adaptations
Theoretical lessons	Use of MS PowerPoint presentations during teaching.	Presentation was shared by Google Meet during teaching.	The use of PowerPoint was very important in online teaching.
	Not applicable	Theoretical sessions were recorded, and after the class, the video was uploaded to the Virtual Teaching Platform which is available to students.	Available sessions and links to other online materials decreased the live attendance in the last third of the course.
	Additional technology is not required for face-to-face interactions.	Interactions with students by chat, microphone, and/or camera.	The use of the camera was a challenge for many students for different reasons (i.e., intimacy, false follow-up of classes, bad Internet connection etc.).
	Using classroom board.	A digital and external board was acquired.	The use of the digital board facilitated many explanations and doubts during teaching (see Figure 1A).
Practical lessons	Students practised and used specific equipment for physical (e.g., wheelchairs), visual (e.g., sound balls), and hearing impairments.	Different teaching strategies were used such as problem-solving activities, discussions, and indirect contact by videos and other simulation tools (e.g., ViaOpta Simulator).	For some specific disabilities, such as sensory ones, simulators play an important role in raising awareness of the collective and an understanding of the activity limitation (see Figure 1B).
Document analysis	Extra readings are provided to students for debates.	The relevant readings are uploaded at the Virtual Teaching Platform.	The lockdown gave more time to students to read and to engage in more academic activities. Better answers in discussions and a higher number of students who followed the extra readings.
Contact with people with disabilities	Normally, with two of the three disability groups.	Direct contact with people with a disability was not possible.	Indirect contact was stimulated with the inclusion of new videos/online resources.
Study case	No differences in the study case assigned (15% of the course) after teaching the contents on Paralympic classification.		
Self-made materials for the group assignment task	Home-made materials for the teamwork-assigned task are part of the proposal to put into practice with people with disabilities (25% of the course).	Students were encouraged to use materials that would be available at their homes.	The quality and complexity of materials were lower compared with the prepandemic group for two reasons: Restrictions in buying or accessing materials for the team proposal. Students had no opportunity to meet and work on the preparation of these materials.
Tutoring	Few hours of tutoring are usually requested during the course by students.	More flexibility for individual tutoring using Google Meet.	The number of tutoring requests increased significantly due to professors’ flexibility and nonrequired physical presence at the office.
Interactive ICTs	Kahoot© and Mentimeter© apps were used to reinforce complex concepts.	The learning games were shared online using Google Meet.	The interaction and implementation of online teaching were more complex.
Complementary activities	Extra (and voluntary) activities are provided to students (e.g., festivals, visit to centers, etc.). Participation in those activities gives students extra scores.	The quarantine provoked the suspension for all these planned activities.	Due to the state of alert, all of the programmed complementary activities were canceled, and students could not take part.
Test	Final exam (60% of the program) comprises 2.5 h. Students can use any type of supporting material during the exam.	The exam was delivered in a Moodle platform instead of handwriting exam. No changes regarding the “open book” policy.	The exam was performed using the Virtual Teaching Campus. Because of the “open book” policy, the exam was configured sequentially (i.e., students must answer questions one-by-one with no options to go back to previous questions already answered). Cheating during tests increased. More professors required for exam delivered in several virtual rooms.

ICTs, information and communication technologies.

### Data Analysis

Self-efficacy scores for the intellectual, physical, and visual subscales are presented as mean and standard deviation. Data were screened for normality of distribution and homogeneity of variance using the Kolmogorov-Smirnov test and Levene’s test, respectively, to determine the appropriateness of using parametric techniques for data analysis. The EA-PEF-AD reliability was assessed by Cronbach’s alpha calculation, considering scores over 0.70 acceptable (Nunnally and Bernstein, 1994). To determine the internal consistency of the scale to evaluate SE, the relationships among EA-PEF-AD subscales were assessed using Pearson’s product-moment correlations (*r*; Reina et al., 2019b). The following scale of magnitudes was used to evaluate correlation coefficients: <0.1, trivial; 0.1–0.3, small; <0.3–0.5, moderate; <0.5–0.7, large; <0.7–0.9, very large; and <0.9–1.0, almost perfect (Hopkins et al., 2009). The pre−/postintervention effects were assessed with a mixed model of repeated measures ANOVA, having the course delivery (i.e., pre- vs. postmeasurements) as the within-group factor and the teaching modality (i.e., prepandemic vs. during pandemic) as the between-group factor. Besides, gender (i.e., male/female), the reported previous training on APA (i.e., yes/no), and the reported previous experiences with PwD (i.e., yes/no) were included in the model as co-variables to explore the potential interaction effect with the main factors of analysis. Practical significance was calculated by partial eta-square (*η_p_*^2^) as a measure of effect size for mean differences with the following interpretation: >0.26, large; between 0.26 and 0.02, moderate; and <0.02, small (Pierce et al., 2004). In addition, pairwise effect sizes (90% of confidence interval) for within-group comparisons are expressed in Cohen’s *d* units and interpreted as follows: trivial (<0.19); small (0.20–0.49); moderate (0.50–0.79); and large (>0.80; Cohen, 1988). All analyses were conducted using the Statistical Package for Social Sciences (version 25.0 for Windows; SPSS Inc., Chicago, IL, United States) and Microsoft Excel (Microsoft, Seattle, WA, United States). The level of statistical significance to reject null hypotheses was set at *p* < 0.05.

## Results

Table 4 shows the reliability scores for the EA-PEF-AD, considering the within-group (i.e., pre- vs. postcourse delivery) and the between-group (i.e., prepandemic vs. pandemic academic years) factors. All the measurements exhibited excellent reliability scores (0.88 < α < 0.96). The scale also shows good internal consistency, with large to very large correlations (0.65 < *r* < 0.80; *p* < 0.001).

**Table 4:** Reliability and internal consistency of the self-efficacy scale.

Self-efficacy subscale	Precourse			Postcourse		
	α	Cor. Ps	Cor. Vs	α	Cor. Ps	Cor. Vs
**Intellectual (Is)**						
Prepandemic	0.89	0.70^**^	0.72^**^	0.88	0.77^**^	0.80^**^
During pandemic	0.92	0.67^**^	0.76^**^	0.93	0.83^**^	0.65^**^
**Physical (Ps)**						
Prepandemic	0.92	–	0.80^**^	0.93	–	0.73^**^
During pandemic	0.95	–	0.74^**^	0.96	–	0.72^**^
**Visual (Vs)**						
Prepandemic	0.93	–	–	0.92	–	–
During pandemic	0.94	–	–	0.95	–	–

α, Cronbach’s alpha; Cor., Pearson’s correlation; Is, intellectual subscale; Ps, physical subscale; Vs, visual subscale.

** *p* < 0.001.

**Figure 2:**
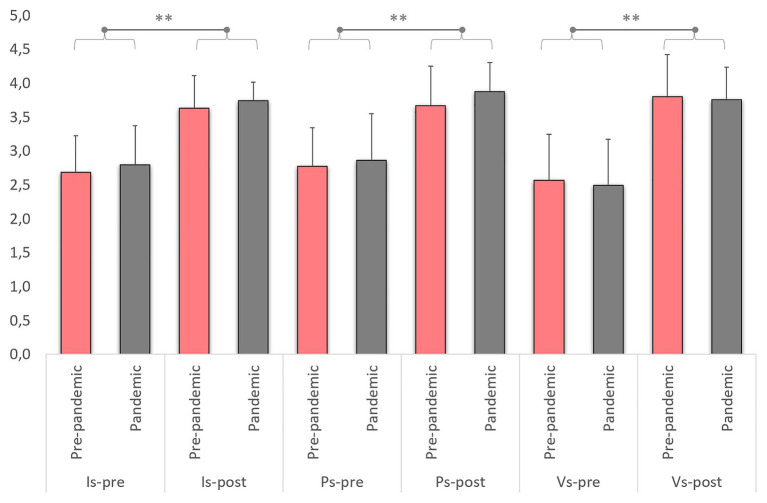
Pre-/postcourse measurements of the three self-efficacy subscales. ^**^p < 0.01.

Figure 2 shows the within-group and between-group main factor comparisons. The ANOVA model revealed overall improvements after the courses for the three subscales: intellectual (*F* = 85.54; *p* < 0.001; *η_p_*^2^ = 0.496, large), physical (*F* = 65.25; *p* < 0.001; *η_p_*^2^ = 0.429, large), and visual (*F* = 123.71; *p* < 0.001; *η_p_*^2^ = 0.587, large) subscales. The prepandemic group reported precourse SE mean scores that ranged 2.6–2.8, and they increase in a 20% of the SE-PETE-D scale (range: 3.6–3.8), that is, large effect sizes for the intellectual (*d* = −1.80, large), physical (*d* = −1.50, large), and visual (*d* = −1.71, large) subscales (*p* < 0.001). Similarly, the group that received two-thirds of the teaching online because of the pandemic reported precourse SE mean scores ranging 2.5–2.8, and they increase up to 3.7–3.9 SE scores. Consequently, this group also improved their perceived SE with large effect sizes in the intellectual (*d* = −1.50), physical (*d* = −1.43), and visual (*d* = −1.86) subscales (*p* < 0.001). On the other hand, there were no differences for the between-group comparisons for any of the three subscales (*p* = 0.194–0.390; *η_p_*^2^ = 0.008–0.019, small).

When exploring the interaction effects between the main factors and the three co-variables introduced in the ANOVA model, no significant interactions were found with gender (*p* = 0.151–0.317; *η_p_*^2^ = 0.012–0.024, small), previous training in APA (*p* = 0.277–0.604; *η_p_*^2^ = 0.003–0.014, small), or previous experiences with PwD (*p* = 0.588–0.774; *η_p_*^2^ = 0.002–0.008, small).

## Discussion

Self-efficacy has been recommended as a preferred theoretical framework for studying the beliefs of prospective PE teachers toward inclusion (Block et al., 2010; Taliaferro et al., 2015). This study aimed to explore the potential impact on SE achievements when teaching suddenly moved to online due to the COVID-19 pandemic, in a sample of university-level students enrolled in a mandatory course on APA for PwD. The lockdown increased the use of information systems and networks to improve distance learning, mainly by using centralized platforms to organize communication processes during instructional activities (Sukendro et al., 2020). To the best of the authors’ knowledge, only the study by Sukendro et al. (2020), in the Australasia region, has explored the effects of COVID-19 on e-learning in university sports sciences students, demonstrating the relationships between the attitude, behavioral intentions, facilitating conditions, perceived ease of use, and perceived usefulness as a core factor for using e-learning by teachers during the pandemic. Practicum in real-education settings (Taliaferro et al., 2015) and observational experiences regarding PE teachers teaching students with a disability (Alhumaid et al., 2020) have been identified as key factors in influencing SE in preservice physical educators. However, these strategies were not possible to apply during the lockdown.

The close link between SE and attitudes toward including PwD in PE has been recently discussed in the literature (Hutzler et al., 2019). According to the systematic review by Lindsay and Edwards (2013), there are four main strategies for improving attitudes and knowledge toward PwD in PE and sports settings, i.e., simulations and/or awareness activities, multimedia interventions, curriculum interventions, and social contact with PwD. These strategies are commonly used in the mandatory 7.5 ECTS course in APA previously described in the “Materials and Methods” section and have contributed to the improvement of SE toward inclusion in in-service PE teachers (Reina et al., 2019a,b). Although the lockdown impeded the real contact with PwD and constrained the opportunities for conducting simulation activities, our study demonstrated similar SE improvements when comparing the pre- vs. postcourse scores in both the prepandemic and during pandemic teaching groups (*p* < 0.001, *d* = large).

With regard to the contact with PwD, McKay (2018) unpacked the components of the Allport (1954) contact theory when conducting awareness activities, which is also applied for improving students’ attitudes (Ocete et al., 2020; Reina et al., 2020) and SE levels in PE teachers (Reina et al., 2019a,b). The four necessary conditions for contact are as follows: (a) equal status, (b) cooperative pursuance of common goals, (c) personal interactions, and (d) identification and acceptance of social norms provided by the authority. Because of the “absent presence” (Varea and González-Calvo, 2020), those components were stimulated with alternative teaching strategies for improving students’ SE. First, equal status was promoted in the assignment task where, in groups, students had to design and stand for an inclusive proposal based on game/recreational activities to be played by university students together with young people with intellectual disability, where all group members participate on equal terms or “equal status.” In other words, they should consider during the task design that an “interaction” between university students and young people with intellectual disabilities would take place. Additionally, all the group members were requested to participate on an equal basis during the explanation of the task as well as the discussion. Second, the cooperation component was also covered through the abovementioned assignment (25% of the total score of the course), requiring collaborative problem-solving (Pöysä-Tarhonen et al., 2018). Besides, the time for this task was increased by 25% of the original time for more profound interactions with the professor. Third, personal interactions with people with intellectual disabilities took place at the beginning of the semester before the lockdown. Those “interactions” would have influenced student’s comprehension about intellectual disability and, therefore, help them to design the assignment task for this population. Fourth, the social norms provided by the authority are present since both professors tried to “guide” the students through an “imagined” contact with PwD, raising questions about potential safety situations; the choice of one material or another; the consideration of adapting certain elements of the task such as rules, space, how to give instructions; etc. Nevertheless, further research would explore the interactions between the abovementioned components of contact (McKay et al., 2017) or when indirect forms of contact are applied as what happens in distance learning.

The effectiveness of direct vs. indirect forms of contact is widely discussed in the systematic review and meta-analysis by Armstrong et al. (2017), concluding that the most effective types of contact appear to be extended (i.e., knowing a fellow “in-group” member has a close relationship with an “out-group member”) and direct contact. However, they did not find clear evidence of the effectiveness for parasocial contact (i.e., being exposed to out-group members through their portrayal using media such as video), and they included only one study using the third form of indirect contact, i.e., guided imagined contact (imagining a positive interaction with an out-group member; Cameron et al., 2011). Both parasocialand guided imagined contact were used for distance learning in this study. Parasocial contact was provided using videos of PwD, including a description of their features and activity limitations derived from the impairment, examples of best practices and interviews, and performance in Paralympic and parasports contexts. All the videos were played in the teaching sessions (i.e., synchronously), but the links were left available to all the students in the virtual teaching platform, something that has been demonstrated as effective for student satisfaction and learning outcomes (Choe et al., 2019). On the other hand, the guided imagined contact was also the main strategy used during the practical lessons, transferred from a sports hall setting to the online platform. The professors agreed on the practical activities per group of disability or parasport (Reina, 2010; Reina et al., 2018) that would benefit this type of indirect contact throughout problem-solving tasks and guided debate (van der Tier et al., 2018). In the online practical lessons, the number of activities decreased but the debates and the student-lecturer interactions increased (McGreevy et al., 2017). In line with this, Kubica et al. (2020) suggested that custom workflows of interactive activities such as several types of polls, question and answer, instant feedback, or group formations with corresponding interactions would be feasible strategies to support interactive activities in livestream lectures.

Simulation was another teaching strategy affected by the confinement. Disability simulation lets people with no disabilities (e.g., physical educators or APA professionals without impairments) to experience and reflect how people with different disabilities experience life (Leo and Goodwin, 2016). According to the course syllabus, students only had face-to-face practical lessons about neurodevelopmental disorders and Special Olympics. They missed the practical lessons and simulation activities about handling physical activity and sports for people with physical, visual, and hearing impairments and the planned Paralympic sports for these populations (i.e., sitting volleyball, boccia, goalball, and five-a-side football). The study by Varea and González-Calvo (2020) explored the perceptions of a group of preservice PE teachers who were keen on undertaking their PE practicum experience, but they were forced to switch to online mode. The preservice teachers concluded that they missed out on an important aspect of their practicum experiences due to the lack of direct contact with students. Additionally, they believed that the subject of PE was losing its identity because of the confinement. Despite our positive SE scores after the course delivery, some testimonies of the students during the institutional quality control for teaching (which was conducted before our postcourse measurement) support this fact: “Despite the online teaching, I liked the way (name of the professor) delivered the classes, but I would have liked to perform the practical sessions face-to-face. In fact, I have found it difficult at times to interpret what they asked for” (student 1) or “The practical sessions have become a bit boring for me as I cannot enjoy them face-to-face; also, if the content of the practical sessions were less theoretical and more practical like how to solve cases as we did in some of the online practices, it would be better” (student 2). This teaching strategy has received some criticism because the insider perspective is omitted from the design, or it assumes that able-bodied individuals acquire meaningful insights into the lives of insiders by participating in short and contrived activities (Leo and Goodwin, 2014). Nevertheless, the changes introduced in distance learning, embracing fewer teaching tasks but with longer discussions in the practical lessons, would have contributed to the students’ SE gains.

At this point, the authors ask why SE scores improved similarly when comparing prepandemic (face-to-face teaching) and pandemic (distance learning) teaching groups. The long experience in APA of the two professors who delivered the course is a factor to consider, since the degree of perceived SE and competence is positively related with students’ outcomes (Goddard et al., 2000). In these pandemic times, a good predictor of higher education students’ acceptance of shifting the educational format is enjoyment, followed by self-efficacy (Rizun and Strzelecki, 2020). Professors used gamification tools such as Kahoot® during the synchronous online sessions to increase learning involvement, a tool that has been successful and resulted in learning gains (Felszeghy et al., 2019). Also, the decision about making available to the students the records of all sessions, together with the links to all the resources used during online teaching, would benefit a more comprehensive understanding and accessibility to the course contents (Meleo-Erwin et al., 2020). Finally, reducing gaps for using technology has been identified as a key factor for teaching PwD and/or delivering APA programs (Ng et al., 2021). In this APA course, all the professors of the same faculty received specific training about the tools used for distance learning, and test trials were also offered to those students who have difficulties with technology use. Altogether, continuing for two-thirds of the syllabus in a distance learning mode did not influence SE gains compared with a teaching group of the previous academic year.

Regarding the co-variables included in the ANOVA analysis, we found no interaction effects with the contextual/demographic variables of students’ gender, previous training in APA, and previous contact with PwD. With regard to gender, this result is in line with the study by Reina et al. (2019a), demonstrating that gender provokes an invariant effect on the SE improvement ratios of in-service PE teacher who participated in an 18-h face-to-face training on inclusive PE, using similar strategies to those of students of this present study. Gender differences concerning SE gains were also not found in another study with a sample of 153 preservice PE teachers (Hutzler et al., 2005). Hence, this present study contributes to the scarce literature regarding this issue (Hutzler et al., 2019). With regard to previous training in APA and contact with PwD, the results from this study are similar to those found with Spanish in-service PE teachers, confirming the invariant effect of demographic variables, such as years of teaching, previous training in APA, previous experience teaching inclusive PE, and teaching setting (e.g., primary or secondary school; Reina et al., 2019a), but also when comparing the professional development program in inclusive PE in insular and peninsular regions (Reina et al., 2019b).

Some limitations should be mentioned. First, this is a natural intervention, where SE assessment is not mandatory for students but helps professors to evaluate students’ gain regarding their perceived competencies for their professional future in APA. Also, the survey fulfillment was carried out voluntarily, and it is possible to have some bias since only those students with a “true” interest in this mandatory course responded to this call. Second, the SE-PETE-D tool includes three vignettes for intellectual, physical, and visual disability cases, respectively. However, the content of the APA course includes other neurodevelopmental disorders such as autism, a wider spectrum of physical impairments (e.g., limb deficiency or brain injury), and hearing impairment. In consequence, the overall learning from these groups of disability may have some transference to the SE measurements in the postcourse assessment. Third, the lockdown in Spain started 3 weeks after the beginning of the second semester, so some content was explained in the regular face-to-face mode. Nevertheless, 12 weeks of the course was online, and the modifications implemented by the professor would help future teaching where the online mode is required or recommendable. Fourth, there is a noticeable heterogeneity across sports sciences and physical activity curriculums in higher education between Spain and European standards for delivering adapted physical activities. The recent research by Ng et al. (2021) explored the preparedness of physical educators to deliver remote adapted physical education using the European Standards in Adapted Physical Activity as a framework to define their competencies. Our study contributes to the literature proposing a set of alternatives and adapted teaching methods for delivering physical activity/education with people with disabilities when teaching must be conducted in remote mode.

In conclusion, distance learning is more complex when subjects have a high percentage of practical content. However, teaching strategies that encourage students’ participation and learning reflections increase the students’ SE regardless of the teaching format (i.e., face-to-face vs. online teaching). The APA course delivered in this study to physical activity and sports sciences university-level students also demonstrates that SE gains are invariant to several demographic co-variables such as gender, previous training in APA, or previous experiences with PwD. Although the teaching methods used in our study would help distance learning formats regarding APA, future research should consider long-term measurements of SE to better understand whether students’ perceptions remain stable. In addition, we provide some guidelines that would stimulate further research for exploring the effects of shorter training programs for professional development or the contributions of different types of indirect contact (i.e., nonface-to-face) with people with a disability in SE gains.

## Data Availability Statement

The raw data supporting the conclusions of this article will be made available by the authors, without undue reservation.

## Ethics Statement

Written informed consent was obtained from the individuals for the publication of any potentially identifiable images or data included in this article.

## Author Contributions

AR and RR performed the data collection, developed the theoretical framework, and interpreted the results. RR performed the analyses and drafted the results, figures, and tables. All authors contributed to the article and approved the submitted version.

### Conflict of Interest

The authors declare that the research was conducted in the absence of any commercial or financial relationships that could be construed as a potential conflict of interest.

## References

[ref1] Alemany-Arrebola I. Rojas-Ruiz G. Granda-Vera J. Mingorance-Estrada Á. C. (2020). Influence of COVID-19 on the perception of academic self-efficacy, state anxiety, and trait anxiety in college students. Front. Psychol. 11:570017. 10.3389/fpsyg.2020.570017, PMID: 33154727 PMC7586314

[ref2] Alhumaid M. M. Khoo S. Bastos T. (2020). Self-efficacy of pre-service physical education teachers toward inclusion in Saudi Arabia. Sustainability 12:3898. 10.3390/su12093898

[ref3] Allen J. Rowan L. Singh P. (2020). Teaching and teacher education in the time of COVID-19. Asia-Pac. J. Teach. Edu. 48, 233–236. 10.1080/1359866X.2020.1752051

[ref4] Allport G. W. (1954). The nature of prejudice. New York: Doubleday Books.

[ref5] Ammar A. Chtourou H. Boukhris O. Trabelsi K. Masmoudi L. Brach M.. (2020). COVID-19 home confinement negatively impacts social participation and life satisfaction: a worldwide multicenter study. Int. J. Environ. Res. Public Health 17:6237. 10.3390/ijerph17176237, PMID: 32867287 PMC7503681

[ref6] Armstrong M. Morris C. Abraham C. Tarrant M. (2017). Interventions utilising contact with people with disabilities to improve children’s attitudes towards disability: a systematic review and meta-analysis. Disabil. Health J. 10, 11–22. 10.1016/j.dhjo.2016.10.003, PMID: 27780687

[ref7] Bandura A. (1991). Social cognitive theory of self-regulation. Organ. Behav. Hum. Decis. Process. 50, 248–287. 10.1016/0749-5978(91)90022-L

[ref8] Bandura A. (1994). “Self-efficacy” in Encyclopedia of human behavior. Vol. 4. ed. Ramachaudran S. (New York, NY, USA: Academic Press), 71–81.

[ref9] Beamer J. A. Yun J. (2014). Physical educators’ beliefs and self-reported behaviors towards including students with autism spectrum disorder. Adapt. Phys. Act. Q. 31, 362–376. 10.1123/apaq.2014-0134 25211482

[ref10] Block M. E. Hutzler Y. Barak S. Klavina A. (2013). Creation and validation of the self-efficacy instrument for physical education teacher education majors toward inclusion. Adapt. Phys. Act. Q. 30, 184–205. 10.1123/apaq.30.2.184 23520246

[ref11] Block M. Taliaferro A. Harris N. Krause J. (2010). Using self-efficacy theory to facilitate inclusion in general physical education. J. Phys. Educ. Rec. Dance 81, 43–46. 10.1080/07303084.2010.10598448

[ref12] Bodde A. E. Seo D. C. (2009). A review of social and environmental barriers to physical activity for adults with intellectual disabilities. Disabil. Health J. 2, 57–66. 10.1016/j.dhjo.2008.11.004, PMID: 21122744

[ref13] BOE (2020). [National Spanish Bulletin], Ministerio de la Presidencia, Relaciones con las Cortes y Memoria Democrática. Real Decreto 463/2020, de 14 de marzo, por el que se declara el estado de alarma para la gestión de la situación de crisis sanitaria ocasionada por el COVID-19. Núm. 67, Sec. I, pp. Pág. 25390–25400.

[ref14] Buffart L. M. Westendorp T. van den Berg-Emons R. J. Stam H. J. Roebroeck M. E. (2009). Perceived barriers to and facilitators of physical activity in young adults with childhood-onset physical disabilities. J. Rehabil. Med. 41, 881–885. 10.2340/16501977-0420, PMID: 19841838

[ref15] Cameron L. Rutland A. Turner R. Holman-Nicolas R. Powell C. (2011). Changing attitudes with a little imagination’: imagined contact effects on young children’s intergroup bias. An. de Psicol. 27, 708–717.

[ref16] Choe R. C. Scuric Z. Eshkol E. Cruser S. Arndt A. Cox R.. (2019). Student satisfaction and learning outcomes in asynchronous online lecture videos. CBE Life Sci. Educ. 18:ar55. 10.1187/cbe.18-08-0171, PMID: 31675279 PMC6829069

[ref17] Cohen J. (1988). Statistical power analysis for the behavioral sciences. Routledge Academic: New York, NY, USA.

[ref61] Del Villar F. (2006). Libro Blanco del título de grado en Ciencias de la Actividad Física y del Deporte [Guidelines for the univesity degree on physical activity and sports sciences]. Madrid: ANECA.

[ref18] Felszeghy S. Pasonen-Seppänen S. Koskela A. Nieminen P. Härkönen K. Paldanius K. M.. (2019). Using online game-based platforms to improve student performance and engagement in histology teaching. BMC Med. Educ. 19:273. 10.1186/s12909-019-1701-0, PMID: 31331319 PMC6647160

[ref19] Gambau-Pinasa V. (2014). Análisis de las salidas profesionales en los planes de estudio de Grado en Ciencias de la Actividad Física y el Deporte en las universidades españolas [analysis of the professional pathways of the degress in physical activity and sports sciences in Spain]. Rev. Española Educ. Física Deportes 405, 31–52.

[ref20] Goddard R. D. Hoy W. K. Hoy A. W. (2000). Collective teacher efficacy: its meaning, measure, and impact on student achievement. Am. Educ. Res. J. 37, 479–507. 10.3102/00028312037002479

[ref21] Griffin M. Smith B. Howe P. D. Phoenix C. (2016). Physical activity among older adults with visual impairment: a scoping review. Kinesiol. Rev. 5, 142–152. 10.1123/kr.2015-0002

[ref22] Hodge S. R. Elliott G. (2013). Physical education majors’ judgments about inclusion and teaching students with disabilities. J. Educ. Train. Stud. 1, 151–157. 10.11114/jets.v1i1.88

[ref23] Hopkins W. Marshall S. Batterham A. Hanin J. (2009). Progressive statistics for studies in sports medicine and exercise science. Med. Sci. Sports Exerc. 41, 3–13. 10.1249/MSS.0b013e31818cb278, PMID: 19092709

[ref24] Hutzler Y. Meier S. Reuker S. Zitomer M. (2019). Attitudes and self-efficacy of physical education teachers toward inclusion of children with disabilities: a narrative review of international literature. Phys. Educ. Sport Pedagog. 24, 249–266. 10.1080/17408989.2019.1571183

[ref25] Hutzler Y. Zach S. Gafni O. (2005). Physical education students’ attitudes and self-efficacy towards the participation of children with special needs in regular classes. Eur. J. Spec. Needs Educ. 20, 309–327. 10.1080/08856250500156038

[ref26] Jaarsma E. A. Dekker R. Koopmans S. A. Dijkstra P. U. Geertzen J. H. (2014). Barriers to and facilitators of sports participation in people with visual impairments. Adapt. Phys. Act. Q. 31, 240–264. 10.1123/2013-0119 25028476

[ref27] Jiménez-Monteagudo L. (2016). *Las competencias en Actividad Física Adaptada del profesional de Ciencias de la Actividad Física y del Deporte: identificación del perfil y efectos de un programa de intervención sobre su mejora* [Competencies on Adapted Physical Activity by professionals in Physical Activity and Sports Sciences]. doctoral dissertation. Madrid: Universidad Autónoma de Madrid. Available at: http://hdl.handle.net/10486/675591 (Accessed January 15, 2020).

[ref28] Kubica T. Hara T. Braun I. Schill A. (2020). “An approach to support interactive activities in live stream lectures” in Addressing Global Challenges and Quality Education conference: 15th European Conference on Technology Enhanced Learning, EC-TEL 2020. eds. Alario-Hoyos C. Rodríguez-Triana M. J. Scheffel M. Arnedillo-Sánchez I. Dennerlein S. M.; September 14–18, 2020 (Heidelberg, Germany: Springer), 432–436.

[ref29] Kudláček M. Morgulec-Adamowicz N. Verellen J. (2010). European standards in adapted physical activities. Olomouc: Palacky University.

[ref30] Kurková P. (2016). Physical activity among older people who are deaf and hard of hearing: perceived barriers and facilitators. Phys. Act. Rev. 4, 72–80. 10.16926/par.2016.04.09

[ref66] Leo J. Goodwin D. (2014). Negotiated meanings of disability simulations in an adapted physical activity course: learning from student reflections. Adapt. Phys. Act. Q. 31, 144–161. 10.1123/apaq.2013-0099 24762388

[ref31] Leo J. Goodwin D. (2016). Simulating others’ realities: insiders reflect on disability simulations. Adapt. Phys. Act. Q. 33, 156–175. 10.1123/APAQ.2015-0031 27078270

[ref65] Lindsay S. Edwards A. (2013). A systematic review of disability awareness interventions for children and youth. Disabil. Rehabil. 35, 623–646. 10.3109/09638288.2012.702850 22831703

[ref32] López-Bueno R. Calatayud J. Casaña J. Casajús J. A. Smith L. Tully M. A.. (2020). COVID-19 confinement and health risk behaviors in Spain. Front. Psychol. 11:1426. 10.3389/fpsyg.2020.01426, PMID: 32581985 PMC7287152

[ref33] McGreevy P. D. Tzioumis V. Degeling C. Johnson J. Brown R. Sands M.. (2017). The use of a virtual online debating platform to facilitate student discussion of potentially polarising topics. Animals 7:68. 10.3390/ani7090068, PMID: 28869501 PMC5615299

[ref34] McKay C. (2018). The value of contact: unpacking Allport’s contact theory to support inclusive education. Palaestra 32, 21–25.

[ref35] McKay C. Park J. Y. Block M. E. (2017). Fidelity criteria development: aligning paralympic school day with contact theory. Adapt. Phys. Act. Q. 34, 233–242. 10.1123/apaq.2017-0064 29542327

[ref36] Meleo-Erwin Z. Kollia B. Fera J. Jahren A. Basch C. (2020). Online support information for students with disabilities in colleges and universities during the COVID-19 pandemic. Disabil. Health J. 14:101013. 10.1016/j.dhjo.2020.101013 33082111 PMC7543900

[ref37] Morley D. Bailey R. Tan J. Cooke B. (2005). Inclusive physical education: teachers’ views of including pupils with special educational needs and/or disabilities in physical education. Eur. Phys. Educ. Rev. 11, 84–107. 10.1177/1356336X05049826

[ref38] Ng K. Reina R. Barrett U. Ferreira J. P. Požėrienė J. Klavina A. (2021). Teachers’ preparedness to deliver remote adapted physical education: updates to the European standards in adapted physical activity. Eur. J. Spec. Needs Educ. 36, 98–113. 10.1080/08856257.2021.1872848

[ref39] Nunnally J. Bernstein L. (1994). Psychometric theory. New York, NY, USA: McGraw-Hill Higher, Inc.

[ref40] Ocete O. Pérez-Tejero J. Coterón J. Reina R. (2020). How do competitiveness and previous contact with people with disabilities impact on attitudes after an awareness intervention in physical education? Phys. Educ. Sport Peda. 10.1080/17408989.2020.1834527 [Epub ahead of print]

[ref41] Pierce C. A. Block R. A. Aguinis H. (2004). Cautionary note on reporting eta-squared values from multifactor ANOVA designs. Educ. Psychol. Meas. 64, 916–924. 10.1177/0013164404264848

[ref42] Pöysä-Tarhonen J. Care E. Awwal N. Häkkinen P. (2018). Pair interactions in online assessments of collaborative problem solving: case-based portraits. Res. Pract. Technol. Enhanc. Learn. 13:12. 10.1186/s41039-018-0079-7 30595740 PMC6294223

[ref43] Reina R. (2010). *La actividad física y deporte adaptado ante el Espacio Europeo de Enseñanza Superior* [adapted physical activity and adapted sports in the European higher education system]. Sevilla: Wanceulen.

[ref44] Reina R. Ferriz R. Roldan A. (2019c). Validation of a physical education teachers’ self-efficacy instrument toward inclusion of students with disabilities. Front. Psychol. 10:2169. 10.3389/fpsyg.2019.02169, PMID: 31632317 PMC6779778

[ref45] Reina R. Healy S. Roldan A. Hemmelmayr I. Klavina A. (2019a). Incluye-T: a professional development program to increase the self-efficacy of physical educators towards inclusion. Phys. Educ. Sport Pedagog. 24, 319–331. 10.1080/17408989.2019.1576863

[ref46] Reina R. Hemmelmayr I. Sierra-Marroquín B. (2016). Autoeficacia de profesores de educación física Para la inclusión de alumnos con discapacidad y su relación con la formación y el contacto previo [self-efficacy of physical education teachers toward inclusion of students with disabilities and regarding their previous training and experiences]. Psychol. Soc. Educ. 8, 93–103. 10.25115/psye.v8i2.455

[ref47] Reina R. Íñiguez-Santiago M. C. Ferriz-Morell R. Martínez-Galindo C. Cebrián-Sánchez M. Roldan A. (2020). The effects of modifying contact, duration, and teaching strategies in awareness interventions on attitudes towards inclusion in physical education. Eur. J. Spec. Needs Educ. 10.1080/08856257.2020.1842973 [Epub ahead of print]

[ref48] Reina R. Roldan A. Hemmelmayr I. Sierra B. (2018). Inclusive physical education and Para-sport. Elche: Limencop S.L.

[ref49] Reina R. Santana A. Montesdeoca R. Roldan A. (2019b). Improving self-efficacy towards inclusion in in-service physical education teachers: a comparison between insular and peninsular regions in Spain. Sustainability 11:5824. 10.3390/su11205824

[ref50] Rice K. L. (2006). A comprehensive look at distance education in the K-12 context. J. Res. Technol. Educ. 38, 425–448. 10.1080/15391523.2006.10782468

[ref67] Rizun M. Strzelecki A. (2020). Students’ acceptance of the Covid-19 impact on shifting higher education to distance learning in Poland. Int, J. Environ. Res. Public Health. 17:6468. 10.3390/ijerph17186468 PMC7558862 32899478

[ref51] Roldan A. Reina R. (2018). “Deporte escolar y universitario en las personas con Discapacidad [sports in schools and university settings for people with disabilities]” in *Libro blanco del deporte de personas con discapacidad en España* [the white book for the sports for people with disabilities in Spain]. eds. Leardy L. Mendoza N. Reina R. Sanz D. J. Pérez-Tejero (Coords.) (Madrid: Cinca), 181–209.

[ref52] Savolainen H. Engelbrecht P. Nel M. Malinen O. (2012). Understanding teachers’ attitudes and self-efficacy in inclusive education: implications for pre-service and in-service teacher education. Eur. J. Spec. Needs Educ. 27, 51–68. 10.1080/08856257.2011.613603

[ref53] Sharma U. Loreman T. Forlin C. (2011). Measuring teacher efficacy to implement inclusive practices. J. Res. Spec. Educ. Needs 12, 12–21. 10.1111/j.1471-3802.2011.01200.x

[ref54] Sukendro S. Habibi A. Khaeruddin K. Indrayana B. Syahruddin S. Makadada F. A.. (2020). Using an extended technology acceptance model to understand students’ use of e-learning during Covid-19: Indonesian sport science education context. Heliyon 6:e05410. 10.1016/j.heliyon.2020.e05410, PMID: 33195843 PMC7644906

[ref55] Taliaferro A. R. Hammond L. (2016). “I don’t have time”: barriers and facilitators to physical activity for adults with intellectual disabilities. Adapt. Phys. Act. Q. 33, 113–133. 10.1123/APAQ.2015-0050 27078268

[ref56] Taliaferro A. R. Hammond L. Wyant K. (2015). Preservice physical educators’ self-efficacy beliefs toward inclusion: the impact of coursework and practicum. Adapt. Phys. Act. Q. 32, 49–67. 10.1123/apaq.2013-0112 25544720

[ref57] UNESCO (2015). Education for all global monitoring report 2015: Education for all 2000–2015 — Achievements and challenges. Paris, France: UNESCO Publishing.

[ref58] van der Tier M. Potting M. Hermans K. (2018). Stimulating the problem-solving abilities of users in an online environment. A study of a Dutch online social casework intervention. Health Soc. Care Community 26, 988–994. 10.1111/hsc.12632, PMID: 30062754

[ref59] van Schijndel-Speet M. Evenhuis H. M. van Wijck R. van Empelen P. Echteld M. A. (2014). Facilitators and barriers to physical activity as perceived by older adults with intellectual disability. J. Intellect. Disabil. Res. 52, 175–186. 10.1352/1934-9556-52.3.175 24937743

[ref60] Varea V. González-Calvo G. (2020). Touchless classes and absent bodies: teaching physical education in times of Covid-19. Sport Educ. Soc. 10.1080/13573322.2020.1791814 [Epub ahed of print]

[ref62] Williams T. L. Smith B. Papathomas A. (2014). The barriers, benefits, and facilitators of leisure time physical activity among people with spinal cord injury: a meta-synthesis of qualitative findings. Health Psychol. Rev. 8, 404–425. 10.1080/17437199.2014.898406, PMID: 25211208

[ref63] Woods A. M. Rhoades J. (2013). Teaching efficacy beliefs of national board certified physical educators. Teach. Teach. 19, 507–526. 10.1080/13540602.2013.827360

[ref64] Zach S. Harari I. Harari N. (2012). Changes in teaching efficacy of pre-service teachers in physical education. Phys. Educ. Sport Pedagog. 17, 447–462. 10.1080/17408989.2011.582491

